# *Mycoplasma hyosynoviae*-associated arthritis in finisher pigs showing signs of lameness in Southwestern Norway

**DOI:** 10.1186/s13028-025-00851-4

**Published:** 2026-01-07

**Authors:** Marit Gaastra Maaland, Borghild Njaerheim Barstad, Marianne Oropeza-Moe

**Affiliations:** 1 Department of Production Animal Clinical Sciences, Faculty of Veterinary Medicine, Norwegian University of Life Sciences, Svebastadveien 116, 4325 Sandnes, Norway; 2 Felleskjøpet Rogaland Agder, Sandvikvegen 21, 4016 Stavanger, Norway

**Keywords:** Antimicrobial use, Bacterial joint disease, Pathology, Swine

## Abstract

Lameness in pigs is a major welfare and economic concern, with infectious arthritis being an important contributor. *Mycoplasma hyosynoviae* is a recognized cause of arthritis in finisher pigs globally, yet its occurrence and clinical relevance in Norway have not been systematically investigated. This small-scale study aimed to elucidate the aetiology of acute lameness in finisher pigs in southwestern Norway through systematic necropsies and microbiological analyses. Between September 2020 and April 2022, ten farms in Rogaland County with recurrent episodes of acute lameness were enrolled. Farm data on housing, lameness history, and antimicrobial use were collected. Thirty-five pigs (mean ± SD weight: 50.7 ± 16.0 kg) were euthanized and subjected to necropsy. Synovial fluid and tissues were sampled for bacterial culture, histopathology, and multiplex PCR (targeting *M. hyosynoviae*, *Mycoplasma hyorhinis*, *Glaesserella parasuis*, and *Streptococcus suis*). The finishing farms produced 384–2,100 finishers annually; lameness typically began 0–3 weeks after arrival. Antimicrobials commonly used included benzylpenicillin procaine and tiamulin. Of 35 pigs examined, *M. hyosynoviae* was detected as the sole agent in 17 cases (16 non-purulent arthritis, 1 serous tenosynovitis) originating from eight farms. Other findings included *Streptococcus dysgalactiae* ssp. equisimilis (*n* = 3), *Trueperella pyogenes* (*n* = 1), and a co-infection with *Actinobacillus pleuropneumoniae* and *M. hyosynoviae* (*n* = 1). In several herds, benzylpenicillin procaine was used despite *M. hyosynoviae’s* intrinsic resistance to beta-lactams. This study provided evidence of *M. hyosynoviae*-associated arthritis in Norwegian finisher pigs after systematic investigation, highlighting the need for diagnostic investigations to guide appropriate antimicrobial therapy. The findings confirmed a common relationship between *M. hyosynoviae* and lameness in finishers pigs and suggest that undiagnosed mycoplasmal arthritis may contribute to welfare and treatment challenges.

## Findings

Lameness in pigs is linked to pain, discomfort, and reduced mobility and represents a significant welfare concern [1, 2]. Lameness also mediates increased veterinary and medical costs, time spent treating sick animals, culling of animals, and reduced weight gain and longer rearing periods for animals that cannot access feed or water due to locomotory problems, affecting welfare, workload, productivity and economy [2, 3]. Common causes of lameness in pigs include non-infectious causes such as osteochondrosis, trauma and fractures, and infectious causes including arthritis caused by bacteria [4]. Bacteria commonly associated with arthritis in pigs include *Mycoplasma hyosynoviae*, *Mycoplasma hyorhinis*, *Erysipelothrix rhusiopathiae*, *Glaesserella parasuis*, *Actinobacillus suis*,* Streptococcus* spp., *Staphylococcus* spp., *Trueperella pyogenes* and *Escherichia coli* [5, 6].

In Norway, “joint diseases in pigs 1–6 months of age” was the second most common health registration in the system Dyrehelseportalen in 2024 [7]. The relative importance of infectious and non-infectious causes of joint diseases has, however, not been systematically investigated. Streptococci, staphylococci, *E. rhusiopathiae*,* G. parasuis* and *T. pyogenes* are perceived to be the most common causes of arthritis in pigs after weaning [8]. To obtain a final diagnosis in cases of lameness, comprehensive and costly diagnostic investigations are often necessary, including transportation of animals for necropsy, bacterial cultivation, and histological evaluation. Polymerase chain reaction (PCR) is the most sensitive method for detection of *Mycoplasma* spp [9]. A final diagnosis in lameness cases is, however, often not obtained, and animals are treated empirically with antimicrobials.

While *M. hyosynoviae* was detected in the lungs of two slaughtered pigs in 1991, arthritis associated with *M. hyosynoviae* was recently documented through routine diagnostic investigations in three farms in Rogaland County, southwestern Norway [10–12]. Mycoplasmas lack a cell wall and are therefore intrinsically resistant to penicillins [13]. In Norway, tiamulin is the drug of choice in confirmed cases of mycoplasma arthritis in pigs after weaning, while benzylpenicillin procaine is the recommended first-line agent for use in other cases of bacterial arthritis [8].

In recent years, field observations showed that several farms in Rogaland experienced outbreaks of acute lameness in finisher pigs, in some cases apparently unresponsive to treatment with penicillin, raising concerns about animal welfare and the adequacy of antimicrobial treatments. It was therefore hypothesized that *M. hyosynoviae*-associated arthritis was related to lameness outbreaks. The aim of this small-scale study was to elucidate the aetiology of acute lameness in finisher pigs by performing systematic necropsies on pigs from farms experiencing recurrent episodes of lameness.

During September 2020 – April 2022, ten farms from six municipalities across county Rogaland were included. Study enrolment was based on a history of acute lameness recurring in several batches of finisher pigs for a period of at least a year. The herds were identified at an information meeting for pig farmers prior to study start, and before and during the study by feed advisers routinely visiting pig herds. A farm visit was conducted, and the farmer was interviewed with standardized questions related to production type, farm size, housing conditions, lameness history and treatment routines. Farmers were instructed to contact the veterinary investigators upon detecting cases of acute lameness in finisher pigs. Pigs that had not been treated with antibiotics and were deemed representative for the farm-specific lameness problem were selected for pathological examination.

All pigs were euthanized on farm and thereafter transported to the Norwegian University of Life Sciences for necropsy. Joints including and proximal to the carpus and tarsus were investigated and sampled according to a standardized protocol including periarticular area, joint capsule, synovial membrane, synovial fluid and joint surfaces. Thereafter, the rest of the pig was necropsied. Skin and soft tissue were removed and the area over each joint capsule flame-sterilized. Synovial fluid was withdrawn using a sterile needle and syringe. If no fluid was recovered, the joint was opened using a sterile scalpel and a sterile inoculating loop was inserted. Sterile sampling of additional organs or tissues was performed if bacterial infection was suspected. Samples were directly inoculated onto blood agar (Difco Columbia blood agar base, Becton, Dickinson and Company, Sparks, Maryland, USA) with 5% sheep blood, a *Staphylococcus aureus* streak as nursing colony, and incubated at 37 °C overnight in 5% CO_2_. Bacterial species were identified by colony morphology, gram staining, CAMP reaction, basic biochemical testing (e.g. oxidase, catalase, indole (BBL DrySlide Indole, Becton, Dickinson and Company) and carbohydrate fermentation), and Lancefield grouping (Prolex Streptococcal Grouping Latex Kit, Pro-Lab Diagnostics, Richmond Hill, Canada) for streptococci. Synovial fluid and/or synovial membrane were collected and frozen for further analysis. The joint samples (in general, two per animal) were shipped to a commercial laboratory (IVD Gesellschaft für Innovative Veterinärdiagnostik mbH, Seelze-Letter, Germany) for analysis by a multiplex PCR including *M. hyosynoviae*, *M. hyorhinis*, *G. parasuis* and *S. suis*. Synovial membrane and joint capsule were collected for histological examination and immersed in 4% formaldehyde. Tissue was thereafter dehydrated in graded ethanol, embedded in paraffin and cut in sections of 3 μm that were mounted on slides before staining with hematoxylin and eosin (HE). Tissue sections were examined by light microscopy. Classification of joint lesions was based on gross pathology and histology. Farm and pig descriptives were analysed using Microsoft Excel.

Nine farms were finisher farms, one was a farrow-to-finish farm. The number of finishers produced per farm and year ranged from 384 to 2,100, with a mean (± SD) of 1,201 (± 557) and a median and IQR of 1,000 and 816, respectively. The finisher farms purchased 30–35 kg pigs from one (eight farms) or two (one farm) regular suppliers. Eight farmers reported an onset of lameness 0–3 weeks after arrival in the finishing unit, of which lameness occurrence lasted for 3–5 weeks (four farmers), throughout the finishing period (one farmer), or unknown (three farmers). Two farmers reported lameness to occur throughout the finisher period with no specific pattern recognized. Five farmers reported the use of benzylpenicillin procaine as first choice in lameness cases, three used tiamulin, and one used amoxicillin (Table 1). One farmer used benzylpenicillin procaine and oxytetracycline interchangeably. All antimicrobials had been prescribed by a veterinarian. All farms had partly slatted flooring systems with sawdust bedding material on the solid concrete floor. Pigs were fed commercial diets which complied with the current recommendations for this age group.

**Table 1:** Choice of antimicrobials and microbial diagnoses on the herd level

	Treatments			Microbial diagnose			
Herd	1st choice	2nd choice	Experienced treatment failure with penicillin	Pigs (n)	*Mycoplasma hyosynoviae*	Others (see Table 2 for details)	Negative / inconclusive
1	Benzylpenicillin procaine	-	No*	4	3	-	1
2	Tiamulin	-	Yes	2	2	-	-
3	Benzylpenicillin procaine	-	No*	5	2	-	3
4	Tiamulin	Benzylpenicillin procaine	Yes	5	4	1	-
5	Benzylpenicillin procaine	-	Yes**	5	-	2	3
6	Benzylpenicillin procaine	Oxytetracycline	Yes	3	1	1	1
7	Benzylpenicillin procaine	Tiamulin	Yes	2	1	-	1
8	Amoxicillin	-	Yes	5	3***	1***	2
9	Benzylpenicillin procaine	-	Yes	3	2	-	1
10	Benzylpenicillin procaine	-	No	1	-	-	1
Total			7 / 10 herds	35	18	5	13

*Farmers in herd 1 and 3 reported approximately 6–7 and 4 days of treatment required for recovery, respectively

**Varying efficacy of penicillin reported

***Coinfection with *Mycoplasma hyosynoviae* and *Actinobacillus pleuropneumoniae* in one pig

A total of 35 pigs were necropsied (Table 1). The number of animals examined by necropsy was limited by available resources, transportation logistics and by the occurrence of clinical lameness during the study period. Mean ± SD weight at necropsy was 50.7 ± 16.0 kg (range: 28–85 kg). Thirteen pigs were female, 22 were castrated male. *M. hyosynoviae* was detected as sole aetiological agent in 17 cases, of which 16 were cases of non-purulent arthritis and one was a serous tenosynovitis (Table 2). *Streptococcus dysgalactiae* ssp. *equisimilis* was detected from three cases of purulent or fibrinopurulent arthritis, one of which was a case of sepsis with polyarthritis and additional detection of the bacterium from the kidneys, spleen, heart, and lungs. *T. pyogenes* was detected in one case of purulent osteomyelitis and arthritis with systemic infection including pneumonic lungs, an abscess, and the liver. Co-infection with *Actinobacillus pleuropneumoniae* and *M. hyosynoviae* was detected in the joint of a pig with purulent arthritis, *A. pleuropneumopniae* was cultivated from chronic pleuropneumonic lungs of the same pig. The 18 pigs that were positive for *M. hyosynoviae* originated from eight different farms, of which seven experienced lameness outbreaks 0–3 weeks after pig arrival.

**Table 2:** Bacteria isolated from various types of arthritis/other causes of lameness in finisher pigs

Pathogen	Non-purulent arthritis	Purulent / fibrinopurulent arthritis	Purulent / fibrinopurulent osteomyelitis and arthritis ± tenosynovitis	Serous tenosynovitis	Other*	Total
*Mycoplasma hyosynoviae*	16			1		17
*Streptococcus dysgalactiae* ssp. *equisimilis*		3				3
*Trueperella pyogenes*			1			1
*Mycoplasma hyosynoviae and Actinobacillus pleuropneumoniae*		1				1
Negative/inconclusive	6	2	2		3	13
Total	22	6	3	1	3	35

*Other: epiphysiolysis (*n* = 1), coronary band abscess (*n* = 1), fibrinopurulent myositis, tenosynovitis and synovitis (*n* = 1)

This study documented the occurrence of *M. hyosynoviae*-associated arthritis in Norwegian farms after systematic investigation. The correlation between non-purulent arthritis and *M. hyosynoviae* confirmed previous reports [2, 14]. *M. hyosynoviae* occur in pig populations across the world and has been described as an important cause of arthritis in finishers in countries such as the US and Denmark [15–17]. The bacterium resides in the tonsils of pigs and may spread hematogenously to the joints [15, 18]. The pain associated with *M. hyosynoviae* arthritis often necessitates therapeutic intervention [2]. In this study, tiamulin and penicillins were used as first choice in three and five herds, respectively, from which pigs were diagnosed with mycoplasma arthritis. The reason for perceived, although somewhat delayed, treatment success in some of the latter farms may have been related to the self-limiting nature of *M. hyosynoviae* arthritis [15]. However, inappropriate antimicrobial use may compromise pig welfare and potentially contribute to antimicrobial resistance development.

Norwegian farmers may slaughter a maximum of 2,100 pigs per year [19]; the studied farms therefore represented a range of production sizes. As this investigation included a limited number of pigs originating from one region and farm selection was based on a history of recurrent lameness, the findings are not necessarily representative of the broader Norwegian pig population. Nevertheless, the study demonstrated the presence of *M. hyosynoviae* in one of the regions with the highest pig density in Norway [20].

In conclusion, *M. hyosynoviae* was identified as a cause of arthritis in pigs in southwestern Norway, underscoring the importance of diagnostics to guide targeted antimicrobial treatment in herds with lameness issues.

## Data Availability

The datasets used and/or analysed during the current study are available from the corresponding author on reasonable request.

## Declarations

### Prior publication

Data have not been published previously.
